# Translational Horizons in the Tumor Microenvironment: Harnessing Breakthroughs and Targeting Cures

**DOI:** 10.1002/med.21338

**Published:** 2015-01-15

**Authors:** Yu Sun

**Affiliations:** 1 Key Laboratory of Stem Cell Biology, Institute of Health Sciences, Shanghai Institutes for Biological Sciences, Chinese Academy of SciencesShanghai, 200031, China; 2 School of Medicine, Shanghai Jiaotong UniversityShanghai, 200025, China; 3 VA Seattle Medical CenterSeattle, WA, 98108; 4 Department of Medicine, University of WashingtonSeattle, WA, 98195

**Keywords:** cancer therapy, acquired resistance, tumor microenvironment, clinical intervention, translational medicine

## Abstract

Chemotherapy and targeted therapy have opened new avenues in clinical oncology. However, there is a lack of response in a substantial percentage of cancer patients and diseases frequently relapse in those who even initially respond. Resistance is, at present, the major barrier to conquering cancer, the most lethal age-related pathology. Identification of mechanisms underlying resistance and development of effective strategies to circumvent treatment pitfalls thereby improving clinical outcomes remain overarching tasks for scientists and clinicians. Growing bodies of data indicate that stromal cells within the genetically stable but metabolically dynamic tumor microenvironment confer acquired resistance against anticancer therapies. Further, treatment itself activates the microenvironment by damaging a large population of benign cells, which can drastically exacerbate disease conditions in a cell nonautonomous manner, and such off-target effects should be well taken into account when establishing future therapeutic rationale. In this review, we highlight relevant biological mechanisms through which the tumor microenvironment drives development of resistance. We discuss some unsolved issues related to the preclinical and clinical trial paradigms that need to be carefully devised, and provide implications for personalized medicine. In the long run, an insightful and accurate understanding of the intricate signaling networks of the tumor microenvironment in pathological settings will guide the design of new clinical interventions particularly combinatorial therapies, and it might help overcome, or at least prevent, the onset of acquired resistance.

## 1. Introduction: Clinical Barriers and Emerging Clues

**Figure 1:**
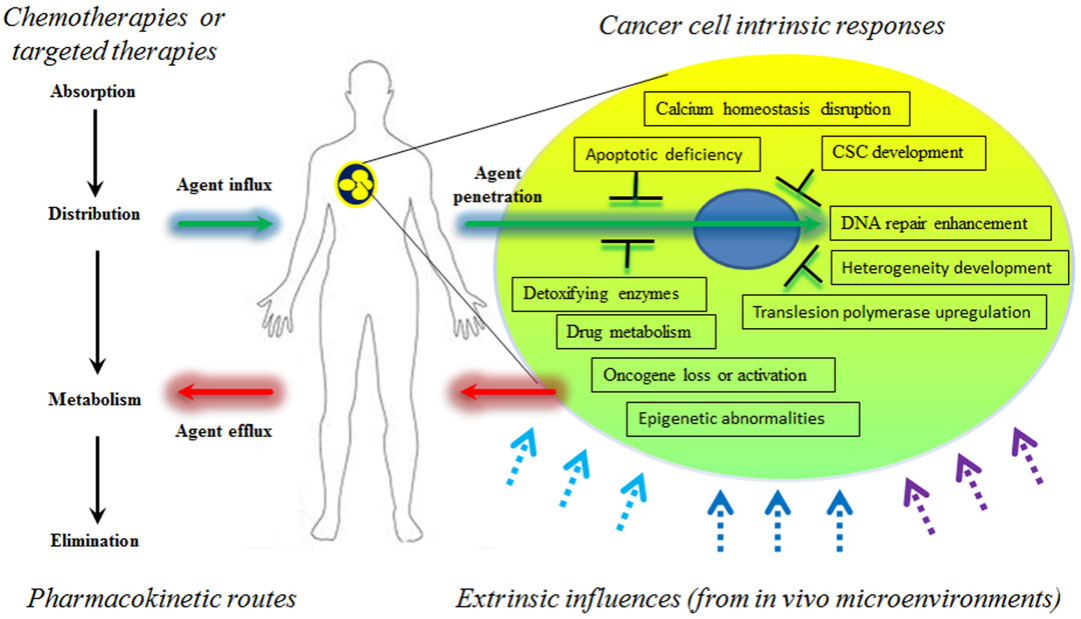
A synoptic paradigm of cancer resistance mechanisms. Resistance to cancer therapies is a major problem facing current clinical oncology. The mechanisms of resistance to classical cytotoxic chemotherapeutics and to therapies designed for selective molecular targets share many features. Upon clinical administration, pharmacokinetic and cell intrinsic factors play important roles in supporting cancer survival, adaptation, and eventually relapse, all are essential steps of resistance phenotype development. However, in response to evolving pathological conditions, oncogenic signals from growing tumors, the tumor microenvironment continually changes over the course of cancer progression, underscoring the need to reconsider its influences as a dynamic process and how tumor drives the construction of its own niche. Bold arrows, pharmacokinetic steps; black text boxes, intrinsic processes occurring in cancer cells during disease progression; dashed and color arrows, factors derived from the neighboring tumor microenvironments that are often activated by various events.

Cancers evolve in complex tissue environments, where they obtain support for expansion, invasion, and metastasis. The past decade has seen significant and accelerated progress in the design, improvement, and application of anticancer therapies; however, most clinical regimens including chemotherapy and targeted therapy ultimately fail to cure patients. Even cancers that show dramatic initial responses to treatments frequently relapse as resistant malignancies, and disease recurrence remains a critical challenge in clinical oncology. The resistance force can arise as a consequence of cell intrinsic changes including upregulation of drug efflux pumps, activation of detoxifying enzymes, increased drug metabolism, loss of specific oncogenes, enhancement of DNA repair machineries including translesion polymerase upregulation, disruption of calcium homeostasis, emergence of apoptotic defects, epigenetic abnormalities, tumor heterogeneity, or plasticity of cancer stemness.^1^–^6^ However, recent data suggest that in addition to innate factors, resistance to cancer therapies can comprehensively result from extrinsic determinants, particularly soluble molecules such as cytokines and growth factors in extracellular environments.^7^,^8^ Further, studies have suggested that rare cancer stem cells (CSCs) are the source of eventual relapse following therapy, as they are usually characterized by increased genomic stability, decreased oxidative stress, or the presence of multiple drug resistance transporters^9^,^10^ (Fig.1).To date, it is well accepted that cancer cells do not expand alone, but evolve through interactions with the surrounding tumor microenvironment (TME).^11^ As key structural and functional components of the TME, resident benign stromal cells regulate the survival, growth, progression, and evolution of solid tumors.^12^ Emerging studies demonstrate that stromal cells synthesize and secrete a large array of soluble factors into the TME niches, as triggering signals delivered in a paracrine fashion, pathologically enabling cancer cells to become therapy resistant.^13^,^14^ Stroma-induced resistance to a multitude of therapeutics is present across various tumor types, as evidenced by experiments with primary cells and cell lines cultured with stromal components isolated from clinical patients or healthy donors. Such resistance is not restricted to conventional cytotoxic or cytostatic agents; rather, it applies to a wide spectrum of chemicals.^15^ Some studies defined the general biological principle of stroma-induced resistance, while other reports substantiated such a phenomenon by extending to even broader range of malignancies including hematopoietic and solid tumors, tumor-stroma interplays, and multiple drug administrations. Stromal cells can protect acute myeloid leukemia cells or chronic lymphocytic leukemia cells against alkylating agents, anthracyclines and nucleoside analogues, mutant Janus kinase 2 (JAK2) cells against JAK inhibitors (or jakinibs), solid tumors such as breast and prostate malignancies against etoposide, doxorubicin, and mitoxantrone, as well as more recently, melanoma against RAF inhibitors such as PLX4720.^7^,^8^,^16^–^18^ Although some components of the stroma can act to restrain the growth of certain tumors,^19^,^20^ mainstream of relevant literatures identified the dominant functions of the microenvironment as a tumor-supportive and resistance-promoting milieu in the course of disease evolution.

## 2. Pathological Implications of the TME

As a major part of the microenvironment, the stroma is a loosely organized scaffold composed of diverse cell types intertwined in an extracellular matrix (ECM), which generates spatial support and mediates cell signaling. In normal tissue the stroma also functions as the physiological barrier against tumorigenesis; however, transformed tumor cells initiate significant changes that can convert the adjacent microenvironment into a TME that supports pathological progression.^21^ In solid tumors, the TME is composed of ECM, carcinoma-associated fibroblasts (CAFs), immune and inflammatory cells, neuroendocrine cells, pericytes, smooth muscle, endothelial cells, and vasculatures.^22^ For hematological malignancies, the TME consists of bone marrow stromal cells (BMSCs), endothelial cells, monocytes, macrophages, osteoblasts, osteoclasts, natural killer (NK) cells, various T and B cells.^23^ The protection provided by the TME allows cancer cells to evade programmed cell death caused by cytotoxic agents and to develop acquired resistance as a fundamental step toward disease relapse. Instigated by extrinsic compartments filled mainly by surrounding stroma, cancers acquire resistance to both targeted therapies and chemotherapy in clinical oncology, a fact that has been well documented and covers multiple mechanisms that implicate derivatives of the stromal cells, a major source, as well as those of the cancer cells, a population that also actively produces factors released into the TME (Fig.1).

### A. Functional Influence of Major Cell Types in the Stroma

As a representative and predominant cell type in the tumor stroma, fibroblasts mainly reside in connective tissue of solid organs, regulating tissue remodeling during wound healing and development.^24^ Enhanced fibroblast growth and activity occurs in morbid contexts, including desmoplasia that is characterized by a dense collagenous stroma and accumulation of fibroblasts within the primary tumor.^25^ Activated fibroblasts express mesenchymal markers, such as fibroblast-specific protein 1, fibroblast-activating protein (FAP), desmin, vimentin, paladin, urokinase-type plasminogen activator receptor associated protein, galectin-3, podoplanin, platelet-derived growth factor receptor, and α smooth muscle actin (α-SMA).^21^ Specifically, myofibroblasts are a type of activated fibroblast with an elongated, spindle-like morphology and enhanced expression of α-SMA.^26^ The emergence of myofibroblasts often accompanies sustained activation of the epidermal growth factor receptor (EGFR) in tumor cells neighbored by desmoplastic environments.^27^ Either the desmoplastic phenotype, presence of myofibroblasts, or increased expression of fibroblastic markers can portend poor outcome. The biological relevance of stromal cells particularly fibroblasts in cancer progression is well established through multiple approaches including miRNA genomic profiling and cotransplantation.^28^–^30^

CAFs are specialized myofibroblasts that exist in pathological settings intimately connected with cancer progression,^31^ and α-SMA can be used as a common marker to identify both myofibroblasts and CAFs. CAFs frequently change the ECM topology, induce stemness, and promote metastasis-initiating cells.^21^,^32^ In particular, CAFs from various tumor types commonly express growth factors, chemokines, and ECM-related proteins, including chemokine (C-C) ligand 2 (CCL2), chemokine (C-X-C) ligand 12 (CXCL12), hepatocyte growth factor (HGF), periostin, and tenascin C.^33^ CAF-derived factors actively regulate cancer survival, promote renewal of CSCs, stimulate inflammation, and enhance angiogenesis, thereby providing a favorable niche for the development of treatment-resistant tumors.^34^ It is not surprising that increased CAF secretion of multiple soluble factors in primary tumors closely correlates with poor prognosis in clinical oncology.^35^

Endothelial cells, pericytes, and smooth muscle cells are stromal lineages that play active roles in angiogenesis and neovasculature development. In the course of tumorigenesis, majority of proangiogenic cytokines are produced by stromal and epithelial cell populations at early neoplastic stages or as a consequence of immune response or local inflammation, while enhanced angiogenesis usually takes place as a late event of tumor progression.^12^ Not limited to adipose tissues, preadipocytes are abundantly present in the bone marrow and stromal compartments of several organs including skin, in which case intradermal adipocytes can mediate fibroblast recruitment upon skin wound healing.^36^ There are functional interconnections between adipose tissues and the upper layers of mesenchymal stroma, the former indeed a pathophysiological source enriched with mesenchymal stem cells as well as cytokines and chemokines, each group capable of inducing stromal cell senescence and increasing cancer cell resistance.^37^–^39^

Under both normal and neoplastic conditions, inflammation involves overall orchestration of multiple cell lineages from the innate and adaptive immune systems. Neutrophils, in most cases, are the first to be attracted to local sites of acute inflammation.^40^ Interleukin 6 (IL-6) and macrophage colony-stimulating factor 1 generated by neutrophils or synthesized by the incipient cancer cells can spur development of myeloid precursors toward the macrophage differentiation.^41^ Macrophages are key players in the immune response, which are bilateral in nature by assuming a phagocytic M1 phenotype to eliminate invasive pathogens and malignant cells, or a permissive M2 feature to prevent immune attack by NK or T cells when the tumor continues advancing after recovery from chemo- or immunotherapy, stimulating angiogenesis and enhancing cancer proliferation, motility, and intravasation.^25^ In cancer biology, the latter type is branded as tumor-associated macrophage (TAM). Particularly, chemokine (C–C) ligand 18 (CCL18) is highly expressed in TAMs and promotes the invasion and metastasis of cancer cells by triggering integrin clustering and enhancing their adherence to the ECM, a phenomenon mediated by the receptor membrane-associated phosphatidylinositol transfer domain-containing proteins (PITPNM3).^42^ Therapeutic success in minimizing the protumoral roles of TAMs in animal models and in early clinical trials suggests that TAMs are attractive targets to prevent resistance as part of combination therapy in cancer treatment.^43^

Macrophages, in particular, can also regulate the inflammatory microenvironment in some pathological settings by generating metabolic products such as reactive oxygen species (ROS), reactive nitrogen species (RNS), cyclooxygenase 2, and prostaglandins, synthesis of which can be targeted pharmacologically.^44^ Suppression of the macrophage product-dependent pathway can both diminish tumor-associated angiogenesis and minimize tumor growth in experimental animals.^45^ Inflammation causes genomic instability through ROS and RNS production, which frequently links inflammation to sporadic carcinogenesis and disease progression.^46^ New studies indicated that hypoxia-inducible factor 1α (HIF1α) promotes tumor production of lactic acid as a by-product of aerobic or anaerobic glycolysis, and subsequently induce the expression of vascular endothelial growth factor (VEGF) and the M2-like polarization of TAMs, allowing these macrophages to play an additive role in tumor growth and treatment resistance.^47^

### B. CSCs and Drug Resistance

As an important part of the cell populations that have malignant potential, CSCs are highly resistant to multiple types of anticancer therapies owing to manifold inherent features, including enhanced aldehyde dehydrogenase activity, assembly of ATP-binding cassette transporter complexes, overexpression of antiapoptotic proteins such as Bcl-2 and Bcl-_XL_, abnormal epigenetic and posttranslational modifications, enhanced DNA damage repair activities, and implication of key prosurvival signaling molecules such as the transmembrane receptor Notch and the nuclear factor-κB (NF-κB).^2^ More importantly, CSCs are relatively quiescent under in vivo conditions, thus less vulnerable to cytotoxic attacks and essentially resistant to most anticancer therapies, which mainly restrain rapidly dividing cells. For instance, second-line resistance to small-molecule inhibitors such as imatinib (Gleevec) has been associated with the persistence of chronic myeloid leukemia stem cells that express BCR-ABL tyrosine kinase enzyme.^48^ Recent studies illustrated the expression of functional EGFR and c-Met in colorectal CSCs that are subject to stimulation by HGF produced abundantly by the stroma. As a line of provided evidence, in patients sensitive to EGFR therapy, administration of c-Met inhibitors fosters CSC eradication and durable tumor regression.^49^ Ironically, some targeted therapies including the antiangiogenic agents sunitinib and bevacizumab even increases CSC expansion. Relevant data revealed that stem/progenitor cell enrichment driven by hypoxia is primarily mediated through HIF1α, and the Akt/β-catenin CSC regulatory pathway is engaged in breast cancer cells upon hypoxic treatments in vitro and in sunitinib-treated xenograft rodents.^50^ Furthermore, the percentage of CAFs that are CD44-positive in tumor tissues is increased after treatment with angiogenesis inhibitors, and such fibroblasts sustain the stemness of cancer stem/initiating cells.^51^ In such a case, CD44 is not only involved in malignant cancer cell drug resistance, but a functional CAF-related molecule contributing to the maintenance of CSC in the TME niche. These reports together highlight that TME-driven CSC advancement compromises the effectiveness of antiangiogenic agents, and imply that to improve clinical indexes, such agents ought to be combined with CSC-targeting pharmaceuticals.

In nature, CSCs are regulated by multiple TME-derived factors including those active in inflammatory cytokine networks, such as IL-1, IL-6, and IL-8.^52^ Expression of signal transducers and activators of transcription 3 (STAT3) mediated by IL-6 is important for maintaining an inflammatory positive feedback loop in breast CSCs.^53^,^54^ A colorectal cancer study disclosed that STAT3 was constitutively activated in CSCs, which were sensitive to STAT3 or IL-6 inhibition.^55^ Furthermore, blockage of CXCR1, an IL-8 receptor, is able to target breast CSCs using specific antibodies or small-molecule inhibitors.^56^

Therapeutic resistance, local recurrence, and distant metastasis remain the major causes of cancer mortality. Dynamic interactions between CSCs and stromal cells represent one of the main mechanisms of treatment resistance and cancer recurrence. In both follicular lymphoma and colorectal cancer, drug-resistant CSCs interact with follicular dendritic cells via CXCL12/chemokine (C-X-C) ligand receptor 4 (CXCR4) signaling to provoke tumorigenicity, and such CSCs are enriched by chemotherapy in the presence of stromal cells.^57^ Thus, integration of CSC-targeting strategy stands a treatment option in future trials for patients after primary therapeutic regimens including standard chemotherapy and targeted therapy.

### C. ECM and Cell Surface Receptors

The structurally dense ECM in tumors has a couple of functions. First, it acts as a physical shield to restrict drug transport, thereby reducing local drug efficacy.^58^ Second, the ECM is responsive to anticancer agents and its composition may change during the treatment, often getting stiffer thereby further limiting drug penetration into the tumors.^21^ Particularly, the synthesis of collagen I and IV increases in breast cancer tissues upon treatment with tamoxifen or neoadjuvant agents, and collagen VI is upregulated in ovarian cancer samples upon cisplatin treatment.^59^–^61^ Conversely, depletion of collagen I in the tumor ECM enhanced drug uptake and caused shrinkage of breast and colon tumors.^62^

On the other hand, the ECM augments drug resistance by providing attachment sites for cancer cells. ECM–cell interactions have consequences for both the cell and the ECM, as binding of cells to ECM not only renders the local ECM stiffer, but dramatically changes the activity of anchored cells.^63^ Termed cell adhesion mediated drug resistance (CAM-DR), such a phenomenon is reported for multiple malignancies including breast cancer, lung cancer, glioma, hepatoma, and leukemia.^64^–^68^ Major proteins of ECM that cause CAM-DR include fibronectin and collagens, both ligands for integrins. In particular, integrin β1 was found to be a key protein mediating CAM-DR, as it binds to a wide range of ECM proteins including collagens, laminins, and Arg-Gly-Asp (RGD) binding ECM proteins including fibronectin.^69^

Integrins are a family of heterodimeric transmembrane receptors, each consisting of an α and a β polypetide chain and bridging for cell–cell and cell–ECM interactions. Once triggered by physical or biochemical stimulations integrins can engage signal transduction pathways most of which eventually induce biological responses such as cell cycle regulation, cell shape recovery, and motility adjustment.^70^ Activation of integrin allows flexible and prompt responses to events at the cell surface, including insults by anticancer agents. Integrin expression is frequently altered in tumor cells including CSC populations, and its upregulation is usually associated with enhanced cancer cell survival, subsequent repopulation, and drug resistance.^71^,^72^ Attachment to the surrounding ECM mediated by integrin can alter responses to chemotherapeutic agents via multiple mechanisms, including but not limited to apoptosis abrogation and drug target modification.^73^

In addition, integrins are involved in signal transduction activities by coordinating the intracellular pathways of transmembrane protein kinases such as receptor tyrosine kinases (RTKs). Although the interplay between integrin and RTKs use to be thought of as unidirectional and supportive, new data indicate that integrins have additional and diverse roles in mammalian cells by regulation of RTK axes via engaging specific adaptors to the cell surface. For instance, β1c integrin recruits Gab1/Shp2 and presents Shp2 to insulin growth factor 1 receptor (IGF-1R), resulting in dephosphorylation of the receptor.^74^ Besides, integrins modulate the PI3K/AKT, Ras/Raf/MEK/ERK, and NF-κB pathways to promote cell survival and enhance drug resistance, suggesting that integrins are likely capable of helping protect cancer cells against multiple targeted agents.^21^ Such activities can be simply exemplified by human epidermal growth factor receptor 2 (Her2) positive metastatic breast cancers in which β1-integrin expression levels were associated with patient responses to the Her2-targeted antibody trastuzumab, a case that led to identification of Her2 as an independent prognostic biomarker for this type of malignancies.^2^,^75^

### D. Soluble Factors in the TME

Within the TME, autocrine and paracrine activation of oncogenic signaling by a large pool of bioactive soluble factors including cytokines, chemokines, proteases, and growth factors derived from the benign stroma or cancer cells have critical roles in generating resistance to both chemotherapy and targeted therapies. As such, soluble factors work as extracellular or intracellular effectors that are able to both trigger and maintain the activation of various survival signaling pathways of cancer, a primary and indispensable step toward development of more malignant phenotypes.

Growth factors bind to RTKs and stimulate both the Ras/Raf/MEK/ERK and PI3K pathways. Stromal secretion of HGF causes phosphorylation of receptor c-Met, engagement of the mitogen-activated protein kinase (MAPK), and implication of PI3K signaling pathways, which culminate in prominent resistance to RAF inhibition.^7^ Simultaneous attenuating pathways activated by RAF and either HGF or c-Met resulted in rescue of drug sensitivity or reversal of drug resistance, implying that combinatorial therapy may be a practical strategy for BRAF-mutant melanoma, and systematic investigation of interactions between cancer cells and their neighboring microenvironments has the potential to disclose critical mechanisms underlying advanced cancer resistance.

Similarly, HGF and c-Met have been linked to drug resistance of Her2-positive, estrogen receptor α-positive, and triple-negative breast cancers (TNBCs). In MCF-7 and T47D breast cancer cells, the acquisition of fulvestrant resistance was accompanied by an increase in c-Met expression, which promoted the response to CAF-secreted HGF.^76^ In TNBCs, CAF-released HGF improves survival of breast cancer cells in the presence of EGFR inhibitors gefitinib and erlotinib.^77^ In Her2-positive breast cancers, HGF is able to restore PI3K and Ras/Raf/MEK/ERK pathway activities that were lost upon treatment with the EGFR/Her2 kinase inhibitor lapatinib, thereby inducing drug resistance.^8^ Several other growth factors also play active roles in cancer resistance. For instance, in lymphoma platelet-derived growth factor C (PDGF-C) secreted by CAFs can counteract the antiangiogenesis therapy by anti-VEGF antibodies, as PDGF-C substitutes VEGF in stimulating capillary outgrowth thus diminishing the treatment efficacy.^78^

It is important to pay attention to another group of proteins secreted by stroma: the cytokines. For example, transforming growth factor (TGF) β has a dual function in cancer biology as it possesses both tumor-suppressive and tumor-permissive potentials. The tumor-promoting effect of TGFβ is mostly seen in the later stages of epithelial cancers, as mainly mediating the induction of epithelial-mesenchymal transition (EMT), which is one of the major driving forces of cancer resistance.^79^ Further, TGFβ is involved in the maintenance of the CSC population, which often shows an increased expression of TGFβ-regulated genes.^80^,^81^ Interestingly, a CSC-enriched gene profile was found after treatment of breast cancer patients with the aromatase inhibitor letrozole or the microtubule toxin docetaxel, suggesting that therapy itself can elicit CSC development.^82^ Altogether, TGFβ is a critical factor produced by the stromal microenvironments and subterraneously contribute to cancer resistance likely through evoking the EMT/CSC axis, which is well established to play long-term roles in tumor evolution.

Stromal cell derived growth factor 1 (SDF-1), or CXCL12, is frequently expressed by TME of multiple tumor types. Specifically, SDF-1 promotes breast cancer growth by binding to its cognate receptor, CXCR4 on cancer cells, rendering ERα-positive breast cancer cells refractory to fulvestrant.^83^ In small cell lung cancer cells that express high levels of CXCR4, SDF-1 promoted integrin-mediated binding to fibronectin and collagen, thereby reducing the sensitivity to chemotherapeutic drugs.^84^ Similarly, IL-1b, tumor necrosis factor-α, and nitric oxide are secreted from inflammatory cells, and induced resistance against etoposide.^85^ By secreting matrix metalloproteinases (MMPs), such as MMP1, CAFs enhanced resistance of head and neck cancer cells to anti-EGFR antibody cetuximab by a yet unknown mechanism.^86^

Chemokine CCL2 has also recently received attention concerning its involvement in the recruitment of infiltrating leukocytes. CCL2 produced by stroma or tumor cells recruits cytotoxic lymphocytes to the TME to suppress or stimulate tumor growth.^87^ This type of dichotomy in the role of chemokines in the immune system is tightly linked to the TME, and depends on the identity of recruited T cells. Similarly, CCL2 is a potent macrophage chemoattractant and is associated with macrophages and tumor stages. Depending on the type of macrophages as M1 or M2, tumor survival and resistance progression might be enhanced.^87^ As supporting evidence, coincubation with a CCL2–antibody enhanced in vitro cell sensitivity to temozolomide, an oral alkylating agent, whereas recombinant CCL2 activated JNK in human melanoma cells and allowed for survival. The combination of a JNK inhibitor with temozolomide was synergistic, and should be more effective due to interference with both tumor and the TME.^88^

For many cancers, increased IL-6 expression plays a central role in augmented chemoresistance. Bone marrow stroma-derived cytokines protect Janus-activated kinase 2-V617F (JAK2(V617F)) mutant cells from the effects of atiprimod, a strong JAK2 inhibitor.^89^ Such protection is associated with high levels of IL-6 and several other factors including fibroblast growth factor and chemokine C-X-C-motif ligand 10 (CXCL10), thus underscoring the importance of targeting the marrow niche in myeloproliferative neoplasms for therapeutic purposes. Long-term treatment of breast cancer cell lines with trastuzumab generates highly enriched CSCs of an EMT phenotype, which allows secretion of over 100-fold more IL-6 than parental cells and promotes resistance development.^90^ Trastuzumab resistance can be mediated by IL-6-involved inflammatory loop, suggesting that blocking such a loop may help overcome resistance to targeted agents. Another recent study demonstrated that p62 expression is reduced in the stroma of several malignancies and particularly, such stromal loss of p62 caused increased prostate epithelial carcinogenesis.^91^ The mechanism, of note, implicates cellular redox regulation through a mammalian target of rapamycin (mTOR) C1/c-Myc pathway of stromal metabolism of nutrients including glucose and amino acid, resulting in elevated stromal cytokine IL-6 production, thereby accelerating tumor progression in the epithelial compartment. Thus, stromal metabolic reprogramming is essential for IL-6-driven epithelial tumorigenesis and treatment resistance.

**Figure 2:**
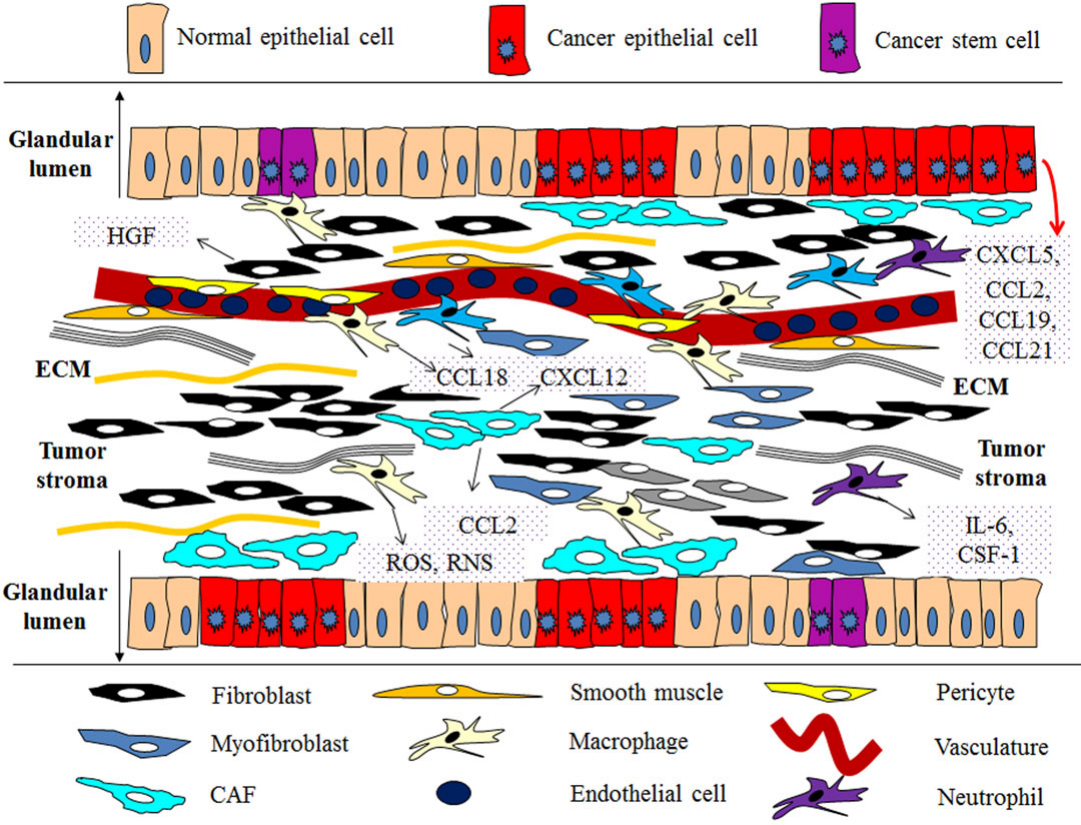
Typical pathological components and signals of the tumor microenvironment. An assemblage of distinct cell types and structural scaffold constitutes most solid tumors such as prostate, lung, and breast cancers. Both the parenchyma and stroma of tumors contain multiple cell types and subtypes that collectively enable tumor growth and progression. Multiple stromal cell types create a succession of tumor microenvironments that change as tumors invade normal tissue and thereafter seed and colonize distant tissues. The abundance, histologic organization, and phenotypic characteristics of the stromal cell types, as well as of the ECM, evolve during progression, thereby enabling primary, invasive, and then metastatic growth. A large array of soluble factors are generated and disseminated into the surrounding milieu, drastically promoting cancer cell survival and stimulate repopulation during the courses of chemotherapy and targeted therapy. Notably, the immune inflammatory cells present in the microenvironment also contribute to therapy resistance by secreting numerous growth factors, cytokines, chemokines that further exacerbate such pathological conditions. ECM, extracellular matrix; CAF, carcinoma-associated fibroblast; ROS, reactive oxygen species; RNS, reactive nitrogen species.

Altogether, the structural and functional presence of various cell types in the TME and their pathophysiologic signals released into TME niches exert comprehensive influences to the biological behaviors of tumors including the priming activities of treatment resistance (Fig.2).

## 3. Impact of Damaged TME to Clinical Therapies

Increasing lines of evidence have recognized noncancerous cells of the TME as major determinants of cancer treatment in a large number of cases, though cancer cell responses are still subject to their intrinsic characteristics. Importantly, data also suggest that novel combination treatments could eventually overcome such “damaged TME” conferred resistance.

**Figure 3:**
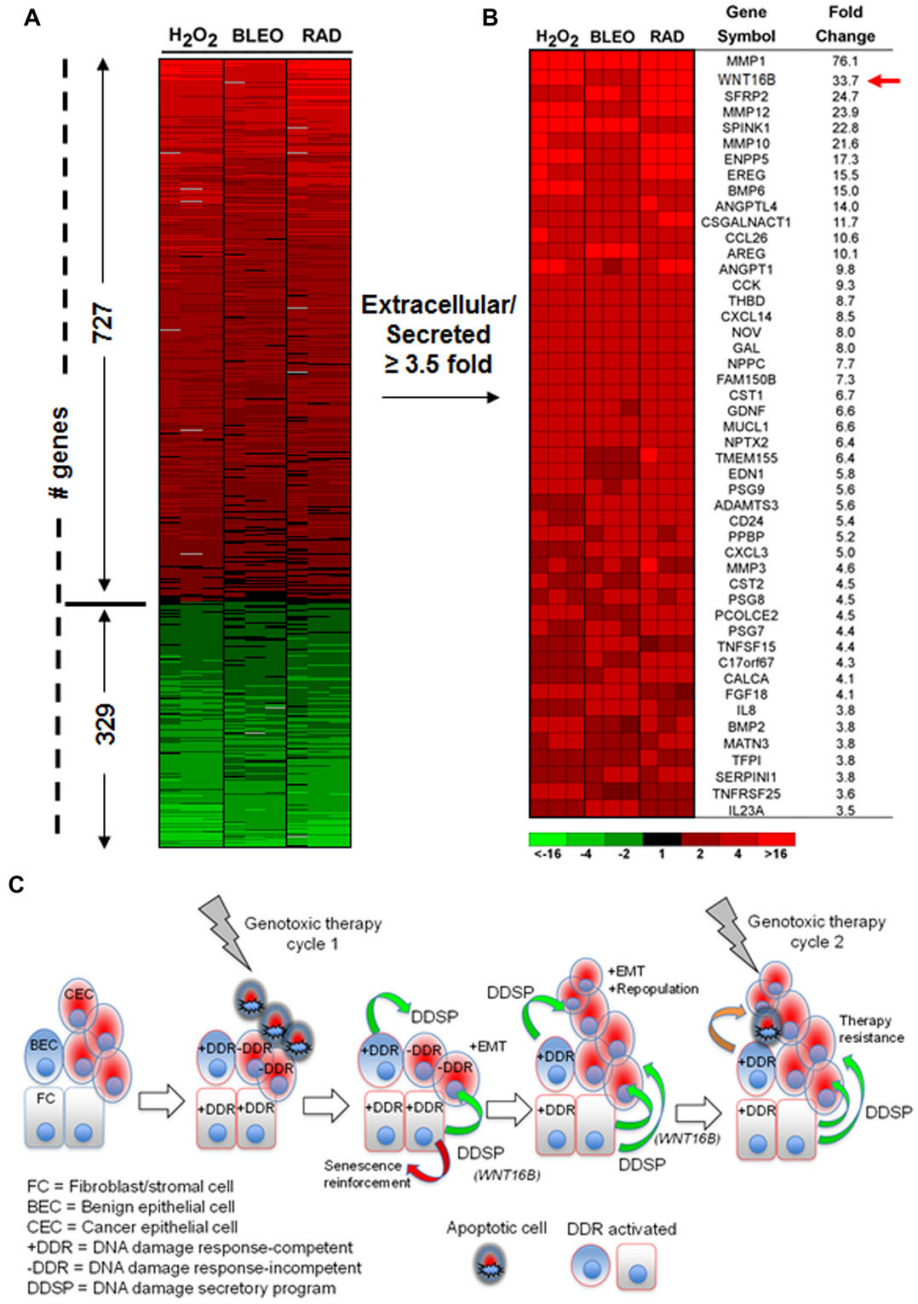
WNT16B is generated upon genotoxic damage to prostate fibroblasts and confers acquired resistance to prostate cancer cells. (A) Bioinformatics analysis of gene expression changes in prostate fibroblasts by microarray hybridization. The heatmap depicts the relative transcript abundance levels after exposure to hydrogen peroxide (H_2_0_2_), bleomycin (BLEO), or ionizing radiation (RAD), agents inducing typical DNA damages. (B) Heatmap and average fold-change measurements of genes annotated as extracellular or secreted factors. Note WNT16B is on the top list of upregulated genes. (C) A model for cell-nonautonomous therapy resistance effects originating in the TME upon genotoxic therapeutics. The initial round of therapy engages an apoptotic or senescence response in subsets of cancer cells and activates a DNA damage response (DDR) in DDR-competent benign cells (+DDR) comprising the TME. The DDR includes a spectrum of autocrine- and paracrine-acting proteins that are capable of reinforcing a senescent phenotype in benign cells and promoting cancer cell repopulation. Paracrine-acting secretory factors including WNT16B promote resistance to subsequent cycles of cytotoxic or targeted therapy. CEC, cancer epithelial cell; BEC, benign epithelial cell; FC, fibroblast cell; −DDR, DDR-incompetent benign cells. Color images adapted from Sun et al.^92^ with permission from Nature Medicine, copyright 2012.

Upregulation of WNT16B by neoadjuvant or regular chemotherapy was found in the TME of prostate, breast, and ovarian cancer patients. Intriguingly, NF-κB was identified as a key signaling node that actively mediates WNT16B production. Cell culture experiments and tumor xenograft models proved the protective effect of fibroblast-derived WNT16B, indicating that WNT16B secreted by stroma attenuates cancer cell apoptosis induced by genotoxicity, and compromises drug response through activation of a DNA damage secretory program (DDSP)^92^,^93^ (Fig.3). The study presents new opportunities for combinatorial treatments, including but not limited to targeting stroma-secreted WNT16B, which would theoretically overcome such a “new” but not “minor” resistance mechanism.^14^,^93^ Besides posing a challenge to conventional dogmas that anticancer treatments mainly restrain cancer itself, the findings raise the novel concept that chemotherapy and radiation induce stroma to promote disease resistance, an important enlightenment that was corroborated by several other simultaneous but mutually independent reports of breast cancer models that strongly imply genotoxicity-incited alterations of the tumor-surrounding stroma as a mechanism that contribute negatively to overall response,^17^,^94^ a pathological facet that should never be overlooked in clinical treatments.

So far studies substantiated the first evidence of WNT16B, supporting that signaling initiated by stroma is actually a critical regulator of cancer response to chemotherapy, targeted therapy, and even endocrine treatment.^95^ Cocultured fibroblasts regulate the in vitro sensitivity of head and neck squamous cell carcinoma to EGFR antibodies or MMP inhibitors.^86^ Further, stromal fibroblast crosstalk plays a crucial role in resistance of lung cancer to EGFR-TKIs through activating the c-Met/PI3K/Akt axis in vitro and in vivo, indicating such an interaction may be a potential therapeutic target for lung cancer patients who carry EGFR-activating mutations.^96^ Similarly, HGF was discovered as one of the central fibroblast-released soluble regulators of in lung cancer sensitivity, and gefitinib combined with anti-HGF antibody or the HGF antagonist NK4 demonstrated excellent efficacy in overcoming the fibroblast-induced EGFR-TKI resistance. A recent report highlighted that the combination treatment of EGFR and c-Met inhibitors with a novel bispecific EGFR/c-Met antibody can block malignant development including resistance in an additive manner compared with treatment with single agents for each pathway.^97^

Years ago, a well-established mouse model of Burkitt's lymphoma was employed to elucidate the mechanism through which paracrine factors in the TME influence lymphoma cell survival following genotoxic chemotherapy. Both IL-6 and tissue inhibitor of metalloproteinase 1 (Timp1) were remarkably released in the thymus upon doxorubicin treatment, a step leading to the establishment of a “chemoresistant niche,” which in turn allowed the survival of residual lymphoma cells as a minimal residue disease and ultimately caused disease relapse.^98^

The growth and maintenance of blood vessels is regulated by multiple pathways, including those mediated by proangiogenic factors secreted by both tumor and stromal cells.^99^ Upon treatments with genotoxic agents, stromal expression of VEGF and other angiogeneic factors including angiopoietin-like 4 (ANGPTL4) and angiopoietin 1 (ANGPT1) is increased, potentially stimulating vasculature development within the damaged TME.^92^,^100^ Simultaneously, synthesis of the secreted frizzled-related protein 2 (SFRP2), a modulator of Wnt signaling, is upregulated in damaged prostate stroma by the chemotherapeutic regime, which can promote angiogenesis via a calcineurin/NFAT pathway in a noncanonical manner.^101^,^102^ There are recent topics about targeting angiopoietin (Ang1, Ang2, Ang4) growth factors that promote accumulation of CAFs and tumor angiogenesis in the TME and TEK (covering Tie1 and Tie2) receptors that control the maturation and plasticity of blood vessels.^103^,^104^ Suppressing angiogenesis in patients to overcome one of the side effects caused by cytotoxic agents is thus a promising strategy to block neoplastic growth and deprive cancer of acquired resistance.

**Figure 4:**
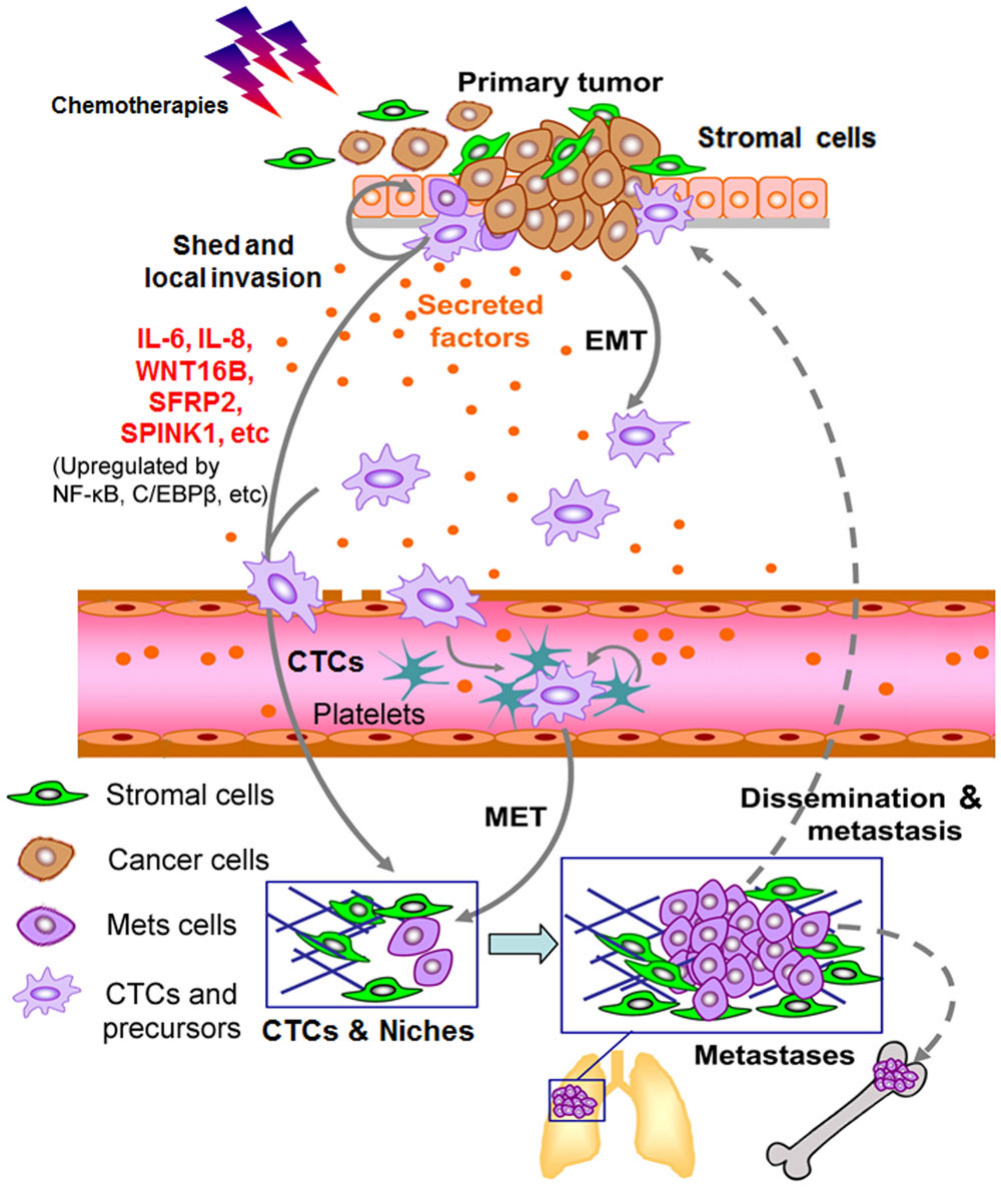
Acquired resistance emerges during anticancer therapies and the long-term consequences include development of circulating tumor cells (CTCs), ectopic metastasis, tumor relapse, and treatment failure. The complex TME is not static, but dynamically responds to a variety of stimuli. Emerging data indicate that chemotherapies particularly genotoxic regimes activate highly conserved damage response programs in benign constituents of the TME. These damage signals, transmitted via master regulators such as NF-κB and C/EBPβ, culminate in a powerful and diverse secretory program DDSP, which generates an activated stroma. Downstream effectors of this program include IL-6, IL-8, WNT16B, SFRP2, SPINK1, and other factors that have been shown to promote adverse cancer phenotypes, among which enhanced resistance is a major and most challenging clinical bottleneck to provide effective cures. The pathological consequence includes initial emerging and subsequent development of CTCs, dissemination of CTCs to multiple distant sites, disease recurrence, and eventual treatment failure. Color image adapted from Kang and Pantel^105^ with permission from Cancer Cell, copyright 2013.

Experimental data of extrinsic factors support that TME dictates the development of chemoresistance by multiple facets including regulation of drug distribution and inflammatory responses. Monitoring tumor status in real time at cellular resolution revealed that myeloid cell infiltration is increased in human breast tumors after chemotherapy and the cellular composition is a strong predictor of overall survival.^94^ Particularly, MMP9 is expressed by myeloid cells and influences vascular leakage and response to doxorubicin. Thus, the response to classical chemotherapeutic drugs can be improved by changing the TME with agents that modify MMP activity and chemokine signaling. Altogether, conventional cancer treatment can be a double-edged sword that is frequently compromised by a therapeutically damaged TME, as a critical step toward development of more advanced disease phenotypes including EMT, generation of circulating tumor cells (CTCs), and dissemination to distant organs^105^ (TableI; Fig.4).

**Table I:** Summary of Anticancer Therapeutics that Are Subject to Resistance Induced by Soluble Factors Released from Stromal (or Cancer) Cells into the TME

Treatment	Cancer type	Targeting mechanism	Source of resistance	Reference
Doxorubicin	Multiple myeloma	Generate DNA intercalation; inhibit topoisomerase II.	Stroma-induced resistance	23
Doxorubicin and pegylated liposomal doxorubicin	Multiple myeloma	Generate DNA intercalation; inhibit topoisomerase II.	Stroma-induced resistance	106
Doxorubicin	Anaplastic thyroid cancer	Generate DNA intercalation; inhibit topoisomerase II.	Stroma-induced resistance; autocrine production of IL-4 and IL-10 promotes thyroid tumor cell progression and resistance to chemotherapy	107, 108
External beam radiation therapy	Anaplastic thyroid cancer	Generate DNA intercalation; inhibit topoisomerase II.	Stroma-induced resistance plays an important role in mortality of thyroid cancer	109
Docetaxel and etoposide	Prostate cancer	Interrupt microtubule depolymerisation/disassembly; causes DNA strand breaks, inhibit topoisomerase II.	IL1R-involved signal axis plays a critical role in the development of chemoresistance in the prostate cancer stem/progenitor cells	110
Mitoxantrone and docetaxel	Prostate cancer	Interrupt microtubule depolymerization/disassembly; generates DNA strand breaks, inhibit topoisomerase II.	Stroma-induced resistance through secretion of multiple soluble factors with WNT16B as a major contributor	92
Vemurafenib ((PLX4032))	BRAF^V600E^-mutant melanoma; BRAF-mutant colorectal cancer and glioblastoma	Interrupts the B-Raf/MEK step on the B-Raf/MEK/ERK pathway.	Resistance to RAF inhibitors is induced by HGF secreted from tumor adjacent stromal cells	7, 8
Ruxolitinib (INCB018424)	JAK2^V617F^-mutant myeloproliferative disorders and high-risk myelofibrosis (a type of bone marrow cancer)	Inhibits Janus kinase inhibitor with selectivity for subtypes JAK1 and JAK2 of this enzyme.	Humoral factors secreted by stromal cells protect myeloproliferative neoplasms clones against JAK2 inhibitor therapy	111
Erlotinib and gefitinib	Metastatic lung, colorectal, pancreatic, or head and neck cancers	Inhibits EGFR, can stimulate apoptosis and differentiation of cancer cell that lack EGFR.	Substantial clinical responses to EGFR tyrosine kinase inhibitors (TKIs) and monoclonal antibodies are now tempered by the increasing number of de novo and acquired resistance mechanisms, the latter contributed by stroma	1
Afatinib	Metastatic nonsmall cell lung cancer, breast cancer, and other EGFR/Her2 driven cancers	Irreversibly inhibits EGFR and Her2 kinases.	Expression of fibroblast growth factor (FGF) 2 and its receptor FGFR1 is upregulated and plays as an escape mechanism for cell survival of afatinib-resistant cancer cells, compensating the loss of EGFR-driven signaling pathway	112

## 4. Development of Advanced Strategies

Although the stromal components of the TME exert profound impacts on disease progression and pose substantial challenges for the advancement of effective therapies, studies addressed the fact that such alterations are indeed creating new opportunities for innovative cancer treatments.

To date there are several herald datasets presenting therapeutic opportunities oriented toward eliminating TEM-induced cancer resistance. In particular, cytokines as well as growth and survival factors released by the TME represent legitimate therapeutic targets. As a major growth stimulator and resistance enhancer by counteracting genotoxic chemotherapy and glucocorticoid-involved treatments typically applied in multiple myeloma, IL-6 is also considered as a therapeutic target in Castleman's disease and several epithelial malignancies including ovarian, prostate, and breast cancer.^16^ According to the studies by Straussman and Wilson, HGF is a key stromal determinant of resistance to BRAF inhibitors, thereby paving the road to investigate combinations of relevant agents with HGF-targeting monoclonal antibodies and/or RTK inhibitors dampening activation of the receptor c-Met.^7^,^8^ Further, identification of the special role of stroma-secreted WNT16B in prostate cancer strongly supports continued translational studies. Of note, antibody therapeutics have taken the mainstay of cancer therapy by either directly targeting specific molecules or serving as optimal vehicles for efficient delivery of cytotoxic chemotherapeutic agents.^113^,^114^ It is appealing to compare the efficacy of specific blockade of WNT16B-engaged pathway (with either antibodies or antibody-conjugated small-molecule inhibitors to target WNT16B itself or the pivotal factors mediating canonical Wnt signaling such as Frizzled, Dishevelled, and β-catenin) and a more general inhibition of the stromal genotoxic response achieved through inactivation of NF-κB complex. Since the NF-κB activity differs among various stromal cell lineages upon exogenous insults like DNA damage, it would be interesting to perform comparative analyses of the effects of NF-κB suppression in the individual cell types isolated from the TME. However, caution should be exercised when designing studies involving NF-κB as a general therapeutic target in cancer therapy, even though numerous convincing experimental data have established NF-κB as a hub regulator of inflammation and a promoter of cancer development. To the contrary, however, new data have indicated that activated NF-κB components promote the sensitivity of cancer cells to agents that induce apoptosis and senescence, thus restraining tumorigenesis.^115^,^116^ As supporting evidence, canonical NF-κB is a Fas transcription activator though the alternative NF-κB is a Fas transcription repressor.^117^ Therefore, NF-κB promotes Fas-mediated cancer cell apoptosis, while undue inhibition of NF-κB may affect Fas-mediated pathway of cell death, eventually interrupting tumor regression mediated by the host immune system.

Adhesion molecules across plasma membrane of cancer cells and/or the underlying stromal cells also make valid target candidates for sensitizing cancer cells to therapeutic agents, provided that treatments by antibodies, peptides, conjugates, or other toxins does not affect normal cells dependent on such stromal cells or surface markers for regular turnover and physiological activities.^16^ Interfering stroma-tumor adhesion or reducing the expression of the adhesion molecules themselves dramatically minimizes the magnitude of stroma-induced cancer resistance.^68^ It is not uncommon that combinatorial therapies targeting both secreted factors and tumor adhesion mechanisms turn out be more effective than strategies targeting the TME alone, a point backed up by several reports. Coculture with BMSCs increases transcriptional levels of diverse oncogenic pathway molecules in multiple myeloma cells, including the HIF1α, Myc, Akt, Ras, NF-κB, human telomerase reverse transcriptase, and Notch pathways.^23^ Second, enhancement of antiapoptotic Bcl-2 family transcripts upon coculture alters BH3 profiling, and the threshold for mitochondria-mediated apoptosis in such cocultured cancer cells is increased, with reduced ability to respond to various treatments.^118^ Furthermore, breast cancer cell attachment to the ECM activates diverse antiapoptotic proteins including EGFR, IGF-1R, and Bcl-2, and induces resistance to dual PI3K/Akt–mTOR inhibitors.^119^ Therefore, stroma-conferred resistance can be mediated by the activation of a wide variety of antiapoptotic signaling axes, where both adhesion-mediated tumor-stroma interactions and TME-secreted factors are at active play. Pharmaceuticals that effectively target both cell–cell communication and extracellular proteins are under intensive bench-top investigation and preclinical trials.

Strategies that effectively inhibit the resistance acquired from the microenvironments confronted by either chemotherapy or targeted therapies have the potential to improve overall therapeutic outcome. Pathological influence of microenvironment on cancer cell survival and subsequent repopulation is usually mediated through the activation of signaling networks that provoke the development of a secretory phenotype and activation of tumor-stroma crosstalk. So far many agents have been developed to inhibit these pathways, including small-molecule inhibitors against specific regulators of key signal pathways, including the p38MAPK cascade, NF-κB complex, CCAAT/enhancer-binding protein components, mTOR molecules, and even DNA damage response moieties; or cytostatic antibodies with the ability to neutralize major soluble factors that play significant roles in advanced cancer phenotypes, such as those targeting IL-6, IL-8, WNT16B, SFRP2, and SPINK1.^93^ A handful of agents acquired FDA approval for the systemic intervention of cancer while many others are in clinical trials. It is not only logical but essential to appraise the use of these agents before, during, or between cycles of anticancer therapies, in order to determine the most effective approach for tumor growth inhibition and therapy outcome improvement. The possible standard therapeutic approach is to consider the disease as systemic at time of diagnosis and to pursue combined therapy employing cytotoxic agents while incorporating feasible cytostatic drugs either concurrently or sequentially, with the latter technically more preferred (TableII). Future continued inputs to perfect screening of candidate anticancer agents will strengthen preclinical pipelines with potential therapeutics that minimize stroma-mediated cancer resistance by acting synergistically with drugs targeting cancer cells in pathological conditions where the TME is actively implicated.

**Table II:** An Example of Optimal Scheduling of Cytotoxic and Cytostatic Therapy to Improve Therapeutic Index in Clinical Oncology

Treatment	Advantages	Disadvantages
Chemotherapy/targeted therapy followed by TME-specific agents	No inhibition of cycle-dependent killing by chemotherapy/targeted therapy	Delayed treatment with active TME-specific agent. No inhibition of repopulation between cycles of chemotherapy/targeted therapy
Chemotherapy/targeted therapy and TME-specific agents given concurrently	Early use of two active therapies. Inhibition of repopulation between cycles of chemotherapy/targeted therapy	Inhibition of repopulation by TME-specific agent might disrupt cycle-dependent killing by chemotherapy/targeted therapy
Short-acting TME-specific agents given between cycles of chemotherapy/targeted therapy and stopped before next cycle	Early use of two active therapies. Inhibition of repopulation between cycles of chemotherapy/targeted therapy. No inhibition of cycle-dependent killing by chemotherapy/targeted therapy	Currently not visible

*Note*. The potential use of TME-specific agents to inhibit repopulation between courses of chemotherapy/targeted therapy is illustrated above by the example of scheduling of adjuvant chemotherapy/targeted therapy (cytotoxic) and TME-selective therapy (can be cytostatic) for patients as advanced stage of solid tumors, such as metastatic castration resistant prostate cancer that is insensitive to androgen deprivation therapy (mCRPC). A logical strategy would include a short-acting cytostatic agent between courses of chemotherapy/targeted therapy to inhibit the repopulation of tumor cells and to stop it before the next cycle so that cells can resume proliferation and be maximally sensitive to subsequent cytotoxic drugs.

## 5. Implications for Personalized Medicine

Among the previously unrecognized implications of tumor–microenvironment interactions, a major one is that development of molecular markers for personalized medicine and their prospective application in clinical oncology will be significantly dependent on the manner and consequence of such “in-milieu” crosstalk. These markers are usually identified and established by linking the cancer cell response to a given therapy with the engagement of oncogenic signaling networks, but new strategies that allow to improve existing therapies and to open new therapeutic avenues by identifying more predictive biomarkers are still expected. As the local microenvironment can influence both the biological profile and treatment responsiveness of cancer cells, it should be integrated into in vitro assays and preclinical studies to identify candidate markers of therapy resistance with the aim to avoid confounding effects while improving their potential use for molecular diagnostics in clinical practice.^120^

To date majority of the proposed molecules for individualized cancer treatments are based on mutations found in particular subtypes of human malignancies, such as TMPRSS2-ERG gene fusions in prostate cancer patients. Many such genomic alterations involve constitutively activated oncogenes or pathways that can be targeted by small-molecule inhibitors available in clinics. For most of these markers, the favorable outcome is usually associated with a higher response rate to a certain treatment, although a large number of cancer patients still failed to respond, indicating the complexity of clinical intervention. Unfortunately, even advanced and exhaustive high-resolution genetic approaches including deep sequencing techniques for a large body of examples were not able to discover additional genomic abnormalities that account for such unresponsiveness. Recent data suggested that in majority of these cases the incompatible genotypes observed against the clinical responses to chemotherapies and/or targeted therapies may be ascribed to stroma-related factors or other TME influences, which cannot be exclusively deduced from the cancer cell genotype alone.^7^,^8^,^15^,^92^ These findings not only heralds continued future research on cancer treatment sensitivity that is significantly susceptible to cancer-neighboring microenvironments, but highlight that technical advancement for high-throughput profiling of this complex pathological landscape is a very important task for cancer research. New lines of evidence linking the TME and cancer resistance suggest opportunities for rational, precise, and individualized combination treatments.

One of the open issues in realizing the new concept of incorporating stroma-derived factors into clinical diagnosis is which markers can be utilized as a gold standard for individual evaluation. It is always the case that patients have response heterogeneity upon treatments, and those who previously underwent anticancer regimens may not show continued clinical indexes, partially due to the chronic phenotypes of damaged cells including senescence, a fine-tuned cellular activity through biological evolution.^121^ Second, how to establish these markers with the currently available methodologies seems to be a question that must be addressed. As several animal models are adapted to more intelligently integrate tumor–microenvironment interactions, they will accelerate the identification of candidate biomarkers for further validation in clinical trials.^23^,^122^,^123^ These attempts would definitely help improve clinical trial designs in order to pre-empt microenvironment-dependent cancer resistance and effectively select for cases that are more likely subject to TME-dependent treatment sensitization. Exploiting such preclinical models to propose candidate markers for validation in clinical trials gains the advantage to bypass the practical challenges of developing biomarkers exclusively through examination with human samples, particularly given that only a small patient subpopulation can respond to a certain targeted therapy. As there are challenges in preparing and analyzing clinical specimens from disseminated metastatic diseases usually caused by CTCs, an extraordinary advantage of orthotopic preclinical models including transgenic animals and site-oriented implantation is that they can be leveraged to identify candidate markers of systemic or local response to a given agent in different metastatic sites. The enhanced expression of several stroma-specific soluble factors such as IL-6, Timp1, WNT16B, SPINK1, SFRP2, and ANGPTL4 upon genotoxic therapies unmasked in recent years makes excellent supplements for such purposes. Third, transgenic and xenografted mice are the most common animal models in cancer biology. However, the average rate of successful translation from rodent models to clinical trials is statistically less than 8%.^124^ In contrast, companion animals with spontaneous neoplasms remain an underexploited tool for translation of investigational anticancer therapies. Companion animals particularly cats and dogs have a relatively high incidence of cancers, with biological features and therapeutic response to cytotoxicity resembling those of humans.^125^,^126^ Shorter lifespan, naturally occurring tumors, and rapidly developed pathologies are among the main advantages of these animals.^126^ Thus, it is compelling to discover molecular targets for prospective therapeutics, which allows to improve survival and quality of life in cancer patients with companion animals as experimental models. Fourth, stromal cell lineages are genetically stable compared to cancer cells and constitute a reliable target for immunotherapy. DNA vaccines against FAP can activate CD8^+^ T cells, specifically kill the CAFs, and increase intratumoral drug uptake.^127^ Once combined with doxorubicin such agents caused tumor regression, as proved by complete tumor rejection in half of the tested animals in contrast to the group that received chemotherapy alone but ended with no survival.^127^ Given the accumulating evidence of tumor heterogeneity, however, whether epigenetic alterations occur in stromal cells particularly those of the potential to impact the stromal-epithelial interactions under therapeutic conditions, remain largely unclear. Further efforts to explore such activities of stromal cells to provide therapeutic options are of high interest in TME biology. Last but not least, what is the best approach to exploit antibody therapeutics in future clinics remains an active topic. Antibody treatment in cancer has not only a rich history but a promising future. Though it remains unclear what new platforms will prove to be more efficacious, it is distinct that multiple novel and advanced methodologies will be explored in the years to come, and interventions composed of antibodies combined with other cytotoxic agents represented by antibody-drug conjugates are rapidly arising. In particular, most of clinically applied monoclonal antibodies in current targeted therapies belong to the human IgG1 subclass, which is more effective in engaging Fcγ receptors on macrophages, neutrophils, and NK cells, thus able to eliminate antibody-bound target cells via antibody-dependent phagocytosis or antibody-dependent cellular cytotoxicity.^113^ Together, novel ideas to therapeutically steer the immune response or the TME are on the dawning horizon of antibody medicine for cancer patients.

The functional role of the TME as an incrementally recognized modulator of treatment responsiveness is emerging just in recent decade. The design and development of novel anticancer therapies will require a comprehensive and insightful appreciation of the pathological significance of the TME as well as an appropriate evaluation of new agents in relevant microenvironment systems that can recapitulate the disease progression in patients. Besides, these systems will accelerate the paces to adapt specific regimens to patient subgroups, which cannot be determined exclusively by the cancer cell innate features but also by biomarkers presenting the characteristics of their surrounding TME milieu. The “orthogonal” large-scale assessment of the molecular features and the therapeutic responsiveness of cancer cells in the TME will be the upcoming frontier for prospective novel therapy identification and their rapid translation to improve clinical outcomes across multiple malignancies.

## 6. Concluding Remarks and Future Perspectives

Current treatment strategies are focused on targeting the cancer cells, but mostly ignore the TME. Multiple types of stromal cells compose a pathologically active and metabolically dynamic niche essential for the development of resistant cancer cells, frequently through paracrine signaling with cancer cells. Despite the daunting challenge of acquired resistance and the complexities caused by tumor–microenvironment interplays, one should admit that targeted therapies and chemotherapeutics are effective in many pathological settings, significantly prolonging the lifespan of patients, or even producing cures in certain cases. Nevertheless, more lessons should be learned from experiences with first waves of conventional cytotoxic treatments and targeted drugs to employ the increasing arsenal of anticancer therapies. Excellent anticancer agent combinations are often proposed by recognition of in vitro and in vivo synergy between these pharmaceuticals; however, dampening a single pathway at multiple points or on separate signaling sections may sometimes provide a relatively simple “escape route” for the cancer, probably the most “cunning” disease that claims numerous lives per year. In contrast, orthogonal therapies that target oncogenic signaling pathways of cancer cells while manipulating released factors or key signal transduction nodes of the activated TME may provide an ideal and optimal option to open the avenues for advanced drug development and clinical administration to thoroughly abrogate cancer resistance in clinical settings. Most importantly, it is necessary to stratify patients according to the likelihood that they may respond to a specific singular treatment or certain combinatorial therapy. It is imaginable that powerful high-throughput techniques such as RNA-Seq, ChIP-Seq, miRNA chips, lncRNA profiling, and next-generation sequencing conveys a large pool of data that can be used to identify predictive biomarkers for therapeutic strategy per patient. On the other hand, a large array of human cell lines is used worldwide but whether they represent the best platform to establish clinically meaningful biomarkers and to appraise compound combinations is a matter of debate. Instead, patient-derived xenograft models that faithfully model patient tumor progression are increasingly applied to allow accurate assessment of disease resistance, evaluation of potential drug combinations, and determination of biomarker values in disease therapeutics. In a long run, preclinical studies should be translated into to the clinics for advanced evaluation, which necessitates the development of “intelligent” practical trials involving cutting-edge-front techniques of molecular pathology and large-scale protein arrays.

This is an exciting era to study how TME distorts cancer therapy indexes in clinically relevant backdrops. With the rapid discovery of multiple new pathways and a myriad of functional components there remain many more questions than answers. Clearly, understanding the biological influence of microenvironments on disease evolution, and the existence of additional pathological factors associated with these conditions is of high public interest and holds research priority. With the use of a combination of neoadjuvant agents and targeted therapies together with more traditional discovery platforms, establishment and advancement of the long-sought preventive, precise, and personalized medicine finally seems within reach.

## Abbreviations

α-SMA: α smooth muscle actin
ANGPTL4: angiopoietin-like 4
ANGPT1: angiopoietin 1
BMSC: bone marrow stroma cell
CAF: carcinoma-associated fibroblast
CAM-DR: cell adhesion mediated drug resistance
CCL2: chemokine (C-C) ligand 2
CCL18: chemokine (C–C) ligand 18
C/EBP: CCAAT/enhancer-binding protein
CSC: cancer stem cell
CTC: circulating tumor cell
CXCL10: chemokine (C-X-C) ligand 10
CXCL12: chemokine (C-X-C) ligand 12
CXCR4: chemokine (C-X-C) ligand receptor 4
DDR: DNA damage response
DDSP: DNA damage secretory program
ECM: extracellular matrix
EGFR: epidermal growth factor receptor
EMT: epithelial-mesenchymal transition
ErbB2 (or Her2): human epidermal growth factor receptor 2
FAP: fibroblast-activating protein
FGF: fibroblast growth factor
HGF: hepatocyte growth factor
HIF1α: hypoxia-inducible factor 1α
IGF-1R: insulin growth factor 1 receptor
IL: interleukin
JAK2: Janus kinase 2
MAPK: mitogen-activated protein kinase
MMP: matrix metalloproteinase
mTOR: mammalian target of rapamycin
NF-kB: nuclear factor kappa-light-chain-enhancer of activated B cells
NK: natural killer
PDGF: platelet-derived growth factor
PITPNM3: membrane-associated phosphatidylinositol transfer domain-containing protein 3
RGD: Arg-Gly-Asp
RNS: reactive nitrogen species
ROS: reactive oxygen species
RTK: receptor tyrosine kinase
SDF-1: stromal cell derived growth factor 1
SFRP2: secreted frizzled-related protein 2
STAT3: signal transducers and activators of transcription 3
TAM: tumor-associated macrophage
TGFβ: transforming growth factor β
Timp1: tissue inhibitor of metalloproteinase 1
TKI: tyrosine kinase inhibitor
TME: tumor microenvironment
TNBC: triple-negative breast cancer
tPA: tissue-type plasminogen activator
uPA: urokinase plasminogen activator
VEGF: vascular endothelial growth factor
